# Magnetic nanoparticles coated with polyarabic acid demonstrate enhanced drug delivery and imaging properties for cancer theranostic applications

**DOI:** 10.1038/s41598-017-00836-y

**Published:** 2017-04-10

**Authors:** Maria Patitsa, Konstantina Karathanou, Zoi Kanaki, Lamprini Tzioga, Natassa Pippa, Constantinos Demetzos, Dimitris A. Verganelakis, Zoe Cournia, Apostolos Klinakis

**Affiliations:** 1 Biomedical Research Foundation Academy of Athens, Athens, 11527 Greece; 2 Department of Informatics and Telecommunications, Postgraduate Program: ‘Information Technologies in Medicine and Biology’, University of Athens, Athens, 15784 Greece; 3 Department of Pharmaceutical Technology, National and Kapodistrian University of Athens, Athens, 15771 Greece; 4 Encephalos-Euromedica Medical Diagnostic Center, Athens, 15233 Greece

## Abstract

Therapeutic targeting of tumor cells with drug nanocarriers relies upon successful interaction with membranes and efficient cell internalization. A further consideration is that engineered nanomaterials should not damage healthy tissues upon contact. A critical factor in this process is the external coating of drug delivery nanodevices. Using in silico, *in vitro* and *in vivo* studies, we show for the first time that magnetic nanoparticles coated with polyarabic acid have superior imaging, therapeutic, and biocompatibility properties. We demonstrate that polyarabic acid coating allows for efficient penetration of cell membranes and internalization into breast cancer cells. Polyarabic acid also allows reversible loading of the chemotherapeutic drug Doxorubicin, which upon release suppresses tumor growth *in vivo* in a mouse model of breast cancer. Furthermore, these nanomaterials provide *in vivo* contrasting properties, which directly compare with commercial gadolinium-based contrasting agents. Finally, we report excellent biocompatibility, as these nanomaterial cause minimal, if any cytotoxicity *in vitro* and *in vivo*. We thus propose that magnetic nanodevices coated with polyarabic acid offer a new avenue for theranostics efforts as efficient drug carriers, while providing excellent contrasting properties due to their ferrous magnetic core, which can help the future design of nanomaterials for cancer imaging and therapy.

## Introduction

Nanoparticles (NPs) have a unique role in the field of nanomedicine because they can be used in an extensive range of applications including disease diagnosis, drug delivery and therapy^1, 2^. Due to their unique properties, NPs have become the locomotive in the field of “Theranostic Nanomedicine”^3^. NPs can be used in existing imaging techniques such as Magnetic Resonance Imaging (MRI) and X-ray Computed Tomography (CT) and in emerging diagnostic methods such as Fluorescence Molecular Tomography and Photoacoustic Tomography^4^. With respect to therapy, NPs are mainly selected for drug delivery applications because of their wide variety in structure, shape, nature and surface coating/functionalization^5^. Polysaccharide-based NPs are preferred due to their excellent biocompatibility properties^6, 7^.

In this study, we investigated polyarabic acid as coating agent in theranostic devices. Polyarabic acid, a major constituent of acacia gum, is a water-soluble polysaccharide molecule containing L-arabinose, D-galactose, L-rhamnose and D-glucuronic acid in the ratio 3:3:1:1 with excellent biocompatibility^8^. We show that iron oxide NPs coated with polyarabic acid and functionalized with Doxorubucin (DOX) show outstanding membrane penetration properties, excellent drug loading and release behavior, minimal *in vivo* toxicity and promising therapeutic potential, while showing comparable contrasting properties with commercial agents in a clinical MRI system. In conclusion, we propose polyarabic acid as an advantageous NP coating in drug delivery and MR imaging applications.

## Results

### Atomistic Molecular Dynamics (MD) simulations to study the NP-membrane interface

**Figure 1:**
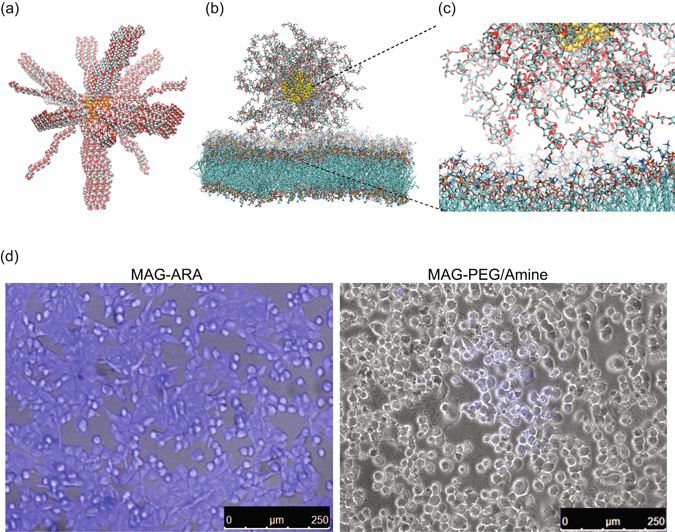
Membrane interaction and internalization of MAG-ARA. (**a**) Initial configuration of the MAR-ARA NP in vacuum. Fe is shown in yellow, oxygen in red, carbon in cyan in van der Waals representation. (**b**) Final configuration of the MAR-ARA NP in contact with a DPPC bilayer. Fe is shown in yellow, oxygen in red, carbon in cyan in van der Waals representation. (**c**) Example of L-arabinose and D-galactose hydrogen bonds as they interact with the phosphate group of DPPC. Hydrogen atoms have been omitted for clarity. Oxygen is shown in red, carbon in cyan in licorice representation. (**d**) Superposition of a confocal fluorescence image (blue) and a bright filed image from MDA-MB-231 breast cancer cells incubated with MAG-ARA and control (MAG-PEG/Amine) NPs.

MD simulations of the polyarabic-coated magnetite nanoparticle (MAG-ARA) were performed in the presence of a DPPC lipid bilayer in order to study the positioning of the nanoparticle in the bilayer, and the interactions of polyarabic acid in atomic-level detail. MAG-ARA was initially placed in the water phase 5 Å away from the lipid bilayer. It is evident from the simulations that MAG-ARA shows a strong preference for the membrane environment and within the first 10 ns it starts interacting with the polar headgroups of the lipid bilayer. However, not all saccharides are equally interacting with the phospholipid headgroups. Quantification of the MD trajectory, by means of hydrogen bonds analyses, showed that MAG-ARA D-galactose and L-arabinose interact preferentially with the phosphate group of the lipid headgroups and perform two hydrogen bonds on average, each (see Figure S1). D-galactose and L-arabinose also interact with the choline group. Such an interaction would imply a ${\rm{CH}}\cdots {\rm{O}}$ bond; indeed, existence of ${\rm{CH}}\cdots {\rm{O}}$ hydrogen bonds is established in the literature^9–11^. This type of hydrogen bond is weaker and has a less directional character than the typical ${\rm{OH}}\cdots {\rm{O}}$ hydrogen bond^12^. However, a recent quantum chemical study of the nature of the ${\rm{CH}}\cdots {\rm{O}}$ interaction has revealed that its strength and directionality qualifies it as a true hydrogen bond^9^. Our quantification shows that indeed D-galactose and L-arabinose each forms one hydrogen bond on average with the choline group (Figure S2). Surprisingly, D-glucuronic acid and L-rhamnose do not interact at all with the lipid headgroups (Figures S1 and S2). Due to the large size of the system (619,500 atoms), simulations could only be performed for 50 ns; although the limited simulation time does not allow for the MAG-ARA internalization to be observed, the membrane-nanoparticle favorable interactions are evident (Figs 1a–c and S1 and S3).

### NPs interaction with lipid membranes

Differential Scanning Calorimetry (DSC) was used to investigate the interactions of Dipalmitoylphosphatidylcholine (DPPC) lipid model membranes with polyarabic acid-coated iron oxide NPs. The main transition temperature, which corresponds to the mobility of the acyl chains of phospholipids, remains unaffected for all samples. The transition enthalpy (ΔΗm) decreased significantly in the DPPC: MAG-ARA 10:1 and 2:1 w/w ratios. According to the phase transition behavior of liposomes, upon heating there is increased head group mobility and water penetration into the interfacial region of the bilayer. We observed strong interactions of MAG-ARA (Figure S4) nanoparticles with the polar groups of DPPC phospholipids, especially at DPPC: MAG-ARA 1:1 and 2:1 w/w ratios.

The cooperativity of the DPPC lipids remained unaffected in the presence of MAG-ARA. The temperature or the pretransition effect decreased and this phenomenology indicates strong interactions between the polar groups of DPPC lipid and the polysaccharide coating of nanoparticle. Additionally, there is a reduction of ΔΗs as the ratio w/w increased, indicating the different hydration forces/interactions between the polysaccharide of NPs and polar groups of DPPC lipid bilayer. A decrease of the pretransition temperature (Ts) indicates that DPPC interaction with the sugar moeities on the polyarabic acid of NPs leads to a thermal energy decrease (enthalpic effect). This decrease of the pretransition temperature is responsible for the reorientation of lipid bilayers from rippled to solid-gel liquid crystal phase (Figure S4).

Dynamic and Electrophoretic Light Scattering (DLS) were used in order to determine the size distribution and the zeta potential of NPs as previously described^13^. MAG-ARA showed near zero z potential 2.4 ± 0.8 mV and a hydrodynamic diameter of 384.5 ± 7.3.

### Cell membrane penetration

In agreement with MD and DSC results, MAG-ARA show excellent cell penetration properties in human breast cancer cells in culture. As indicated in Fig. 1d, blue fluorescent MAG-ARA show a uniform intracellular distribution after 8 hrs incubation with cells, while control MAG-PEG/Amine NPs, in which the coating is composed of PEG_20000_ (Polyethylene glycol 20000 Da) fail to penetrate cell membranes. Previous reports indicate that long PEG chains inhibit cell penetration^14^.

### DOX loading and release

DOX was loaded onto MAG-ARA through interaction of an activated carboxyl group and an amino group on DOX. Loading was performed in neutral pH while release was achieved through exposure of NPs to pH 5.3 similar to what is expected within tumor cells. The efficiency of DOX loading (45%) and release (70%), was measured as a decrease and increase, respectively, in the optical properties of supernatant.

### *In vitro* efficacy and toxicity study

**Figure 2:**
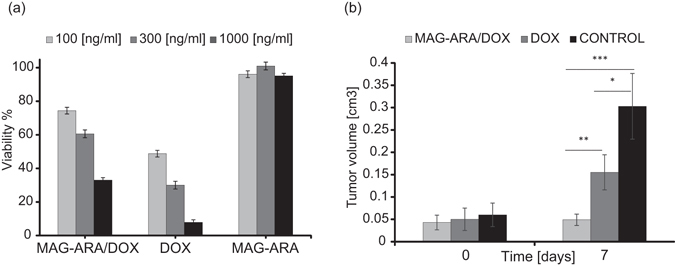
Biological properties of MAG-ARA/DOX NPs. (**a**) *In vitro* toxicity and efficacy of MAG-ARA/DOX NPs (MAG-ARA/DOX) in comparison to soluble DOX (DOX) and nonfunctionalized MAG-ARA NPs in three different concentrations. Experiments were performed in MDA-MB-231 cells and viability was assessed with MTT colorimetric assay. Viability is calculated as percentage of absorbance of untreated cells. (**b**) Bargraph indicating tumor volumes of MDA-MB-231 xenografts treated with MAG-ARA/DOX, soluble DOX (DOX) and vehicle (control), at the time therapy commences (Day 0) and 7 or 14 days later. MAG-ARA/DOX NPs used in these experiments were also coated with FA-PEG2000. *Designates P value < 0.05 and ***designates P value < 0.001.

All *in vitro* experiments were performed in the human breast cancer cell line MDA-MB-231. Therapeutic efficacy was assessed in comparison with soluble DOX. As indicated in Fig. 2a, MAG-ARA/DOX showed comparable effect with soluble DOX on cell growth. Similar experiments with nonfunctionalized MAG-ARA showed marginal cytotoxicity in MDA-MB-231 (Fig. 2a), indicating that the reduced cell numbers observed with functionalized NPs is the result of DOX release and subsequent inhibition of DNA replication rather than nonspecific NP cytotoxicity. Our results demonstrate that MAG-ARA NPs proved effective against cancer cells while showing great biocompatibility.

### *In vivo* experiments

MAG-ARA/DOX NPs were further evaluated for toxicity *in vivo*. While *in vitro* toxicity and efficacy of MAG-ARA and MAG-ARA/DOX NPs, respectively, was not affected by an extra coating with PEG_2000_ (not shown), *in vivo* experiments were performed with NPs further coated with PEG_2000_, which in this particular case was decorated with folate acid (FA). Low molecular weight PEG is known to reduce aggregation and protect NPs from opsonization, while FA, highly expressed by certain tumor cells, facilitates tumor cell recognition^15, 16^. Wild-type mice injected with NPs were biopsied 7 days upon NP administration. Macroscopic observation of injected mice failed to detect any sign of compromised health, while microscopic histological examination of major tissues including the liver, lungs, kidneys and spleen showed no signs of NP aggregation, tissue inflammation or other symptom of toxicity (not shown).

Therapeutic efficacy was assessed *in vivo* in immunocompromised NOD/SCID mice grafted with MDA-MB-231 cells. Tumor volume was calculated based on caliper measurements and treatment commenced (Day 0) when tumors reached a volume of approximately 0.05 cm^3^, at which point vehicle, soluble DOX or MAG-ARA/DOX were injected through the tail vein. On day 7, MAG-ARA/DOX-treated mice already had a statistically significant therapeutic advantage over vehicle and DOX-treated counterparts. After a second dose on Day 7, mice were monitored through Day 14 at which point the experiment was terminated. Similarly to Day 7, MAG-ARA/DOX-treated mice displayed smaller tumor size (Fig. 2b). Beside smaller tumor size, these mice presented a better overall survival with 29/29 mice being alive on Day 14, while only 7/28 and 16/24 treated with vehicle and DOX, respectively survived through the end of the experiment. Survival was independent of initial tumor size on Day 0 (not shown). While vehicle-treated mice presumably died of tumor complications, we cannot exclude the possibility that DOX-treated animals died of a combination of drug toxicity and tumor burden. Our results clearly indicate that MAG-ARA/DOX, while not toxic, deliver sufficient DOX to contain tumor growth. The combination of reduced toxicity and increased efficacy of MAG-ARA/DOX NPs can only be explained by the excellent cell penetration properties of the polyarabic coating in combination with the highly biocompatible nature of the NPs, facilitated by PEG_2000_, and the FA-mediated tumor cell recognition.

### MAG-ARA NPs as contrast agents in MRI

**Figure 3:**
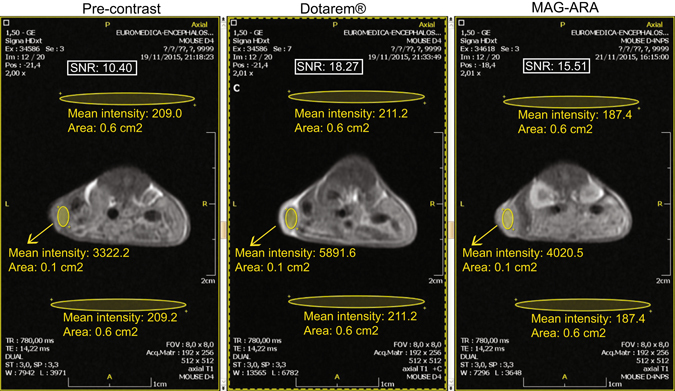
T1 sections of NOD/SCID mice grafted with MDA-MB-231 cells which were MRI-scanned before (Pre-contrast) and after intravenous administration of a commercial contrasting agent (Dotarem) or MAG-ARA NPs. SNR: signal-to-noise ratio. Oval areas represent ROIs used for SNR calculation.

To investigate whether MAG-ARA NPs can be used as imaging agents within the field of a clinical MRI, dispersions of MAG-ARA and the commercially available contrast agent Dotarem were injected into tumor-bearing mice and visualized in a General Electric Signa HDxt 1.5 Tesla clinical MRI. Regions of Interest (ROI) were selected and signal-to-noise ratio (SNR) calculations were performed (Fig. 3). Based on SNRs, contrast-to-noise ratios (CNR) pre- and post-contrast calculated values were 7.87 and 5.11, for Dotarem and MAG-ARA respectively, indicating that MAG-ARA NPs can be used as contrasting agents with comparable to Dotarem results.

## Discussion

In this manuscript, we investigate the potential of polyarabic acid as a coating agent in ferrous magnetic nanoparticles used as drug carriers and contrasting agents for cancer theranostics. We employ both computational and experimental approaches to show that magnetic NPs coated with polyarabic acid optimally interact and efficiently penetrate cell membranes, and internalization was experimentally verified through imaging techniques. Our *in vitro* and *in vivo* experiments indicate that NPs coated with polyarabic acid are biocompatible causing minimal if any cytotoxicity. Further functionalization with PEG_2000_ allowed to decorate NPs with the chemotherapeutic drug Doxorubicin.

Our *in vitro* and *in vivo* results indicate that functionalized NPs can be used as efficient drug carriers while providing excellent contrasting properties due to their ferrous magnetic core.

Polyarabic acid is a polymer with excellent biocompatibility^8^. This is the first time that membrane interaction and internalization of magnetic NPs coated with polyarabic acid are computationally and experimentally studied. Our results show that, besides its well-established biocompatibility, polyarabic acid interacts with lipid bilayers and efficiently penetrate cells. In addition, it provides free groups (e.g. carboxyl groups) that can be exploited for further functionalization. Although further experimentation is warranted, our results indicate that polyarabic acid could be an optimal coating agent for nanodevices in drug delivery and theranostics applications.

## Methods

### Materials

The following commercially available materials were used: Doxorubicin hydrochloride (DOX) and MES (Fisher Scientific); N-(3-Dimethylaminopropyl)-N-ethylcarbodiimide hydrochloride (EDC) and N-hydroxysuccinimide (NHS) (Sigma); Folate-PEG/Amine (PEG = 2000 Da) (FA-PEG_2000_/Amine) (Nanocs); FluidMAG ferrofluids (Chemicell); fluidMAG-ARA consist of an aqueous dispersion of magnetic iron oxides with diameter of spherical magnetic nanoparticles, which are composed of a magnetic magnetite (Fe_3_O_4_) core, a variety of passivation coatings and functional groups depending on the nanoparticle case. The blue fluorescence-labeled nano-screenMAG-ARA and nano-screenMAG-PEG/Amine (PEG = 20000 Da) are as above and carry a blue fluorescence label (excitation/emission: 378/413 nm).

DPPC (1,2-dipalmitoyl-sn-glycero-3-phosphocholine) was purchased from Avanti Polar Lipids Inc. and used without further purification. Mice were anaesthetized with Avertin (Sigma-Aldrich). Injectable Gadoteric acid Dotarem, (Guebert LLC) was used as contrasting agent.

### Modeling and construction of the functionalized magnetic nanoparticle (MAG-ARA)

Computational approaches were used for the modeling and construction of functionalized magnetite (Fe_3_O_4_) nanoparticles (MNPs). Initially, the unit cell of the magnetite crystal was downloaded from the crystallography open database^17^ with CIF number 1011032^18^ and replicated three times in the xyz direction by using the buildCluster module of MAPS^19^. The growing planes of the Fe_3_O_4_ crystal, which correspond to its minimum surface energies, were then used to create the preferred shape of the nanoparticle (NP)^20^. The planes that dominate the magnetite morphology are the (001), followed by the (111) surface, which truncates the corners of the cube^20^. A Wulff crystal morphology^21^, which represents the core of the Fe_3_O_4_ NP, was derived dynamically with our in-house implementation using the lowest surface energies for each Miller index. Its calculated equilibrium morphology is then expressed as a cubic shape of 3-nm diameter with truncated corners (cuboctaedron)^20^.

For functionalizing the magnetite core, polyarabic acid (ARA) was constructed, which is a branched polysaccharide consisting of L-arabinose, D-galactose, L-rhamnose and D-glucuronic acid in the ratio 3:3:1:1 (MAG-ARA). A dynamic code implementation allowed the ligand chains to be attached to selected surface octahedral Fe atoms of the MNP core with the option for the user to define the length of the chains used. The MAG-ARA nanoparticle was covered with 64 ARA chains having a grafting density of 3 chains/nm^2^ with a length of each chain to be 6.5 nm. Subsequently, a DPPC lipid bilayer was built and used to model the cell membrane using the charmm-gui platform^22^ with a tetragonal crystal type and 489 number of lipids on each side of the membrane. The total size of the system including water molecules is 619,500 atoms.

### Atomistic MD simulations

Molecular simulations can help rationalize experimental findings by providing a microscopic-level description of the NP-membrane interactions^23–25^. Atomistic MD simulations were performed in order to assess the interactions between MAG-ARA and the lipid bilayer in atomic-level detail.

The MAG-ARA was placed in the water phase of the lipid bilayer system and atomistic MD simulations were performed in order to describe the nanoparticle-membrane interactions in atomic-level detail. All simulations were carried out using the GROMACS simulation package, version 5.0.6^26^. The topology files for the system were constructed using our in-house code, which included partial charges for magnetite and ligands, and combinations of bonds, angles and dihedrals of the system atoms. The CHARMM36 force-field^27^ was used, with the non-bonded epsilon parameter for van der Waals interactions added for magnetite Fe^2+^ atoms^28^. For the modeling of the water molecules, the TIP3P model was employed^29^. Following minimization and equlibration of the system, a production MD run was performed for 50 ns. The full protocol of the MD simulations can be found in the Supplementary Information.

### Surface modification

Ferromagnetic nanoparticles used in this study have the following components: i) an iron magnetic core, ii) the chemotherapeutic drug DOX and, iii) PEG coating, which ensures protection from the immune and limits aggregation.

For functionalization with DOX and PEG_2000_, 25 μl of magnetic NPs stock solution (25 mg/ml) suspended in 1.5 ml MES solution (pH 5.5) were mixed with 10 mg EDC and 10 mg NHS for 2 hours by shaking at 650 rpm to activate the carboxylate groups. NPs were washed 3 times with distilled de-ionized water to remove free molecules. DOX (0.6 mg) and FA-PEG_2000_/Amine (0.6 mg) were stirred with NPs in 1 ml H_2_O for 18 h at room temperature. NPs were washed several times with phosphate buffered saline (PBS) until the supernatant became colorless. All washes were performed using magnetic separation of NPs. Following drying at room temperature in a rotary evaporator, NPs were stored in 1 ml neutral PBS (pH 7.4). The above procedure refers to preparation of a single mouse dose, injected in a volume of 150 μl and was scaled up for larger preparations. The amount of NPs used was selected based on loading efficiency (see below) and NP toxicity based on the literature^30^. For drug release, DOX-loaded NPs were switched to 1 ml of acidic PBS (pH 5.3). NPs and DOX were incubated at 37 °C for 36 hours on an orbital shaker at 640 rpm. NPs generated from this process were used in *in vitro* and *in vivo* experiments and are referred to throughout the text as MAG-ARA/DOX.

Loading and release efficiency was calculated as follows: following magnetic separation of NPs, 5 uL drops of the supernatant were collected in the beginning and the end of the loading process, and photographed under the N2.1 red fluorescence filter of a LEICA DMRA2 upright microscope. Fluorescence intensity was quantified using the ImageJ software. A reduction of fluorescence was consistently observed as a result of DOX loading onto the NPs. Loading efficiency was calculated by the signal difference between the two measurements. For drug release measurements, loaded NPs were incubated in acidic pH 5.3 and fluorescence intensity was measured as above.

### *In vitro* and *in vivo* testing

To assess *in vitro* cytotoxicity and therapeutic efficacy, human breast cancer MDA-MB-231 cells were incubated for 72 hours with native MAG-ARA as well as MAG-ARA/DOX. Cells were grown on Dulbecco’s Modified Eagles Medium (DMEM) supplemented with 10% fetal bovine serum (FBS) as previously described^31, 32^. Cell viability was measured using the colorimetric MTT assay. DOX solution was used as positive control for efficacy. For *in vivo* toxicity studies, equal amounts of MAG-ARA as well as MAG-ARA/DOX were injected through the tail vein into 2 month-old wild-type C57Bl/6 mice. Following intravenous injection, mice were monitored for 1 week before been sacrificed. Female NOD/SCID mice were purchased from the Jackson repository and housed in individually ventilated cages under specific pathogen–free conditions in full compliance with FELASA (Federation of Laboratory Animal Science Associations) recommendations in the Animal House Facility of the Biomedical Research Foundation of the Academy of Athens (BRFAA, Greece). All experiments were performed in accordance with national and international guidelines and regulations and were approved by the Institutional Committee on Ethics of Animal Experiments and the Greek Ministry of Agriculture. All major organs were dissected, fixed in 10% formalin and processed for paraffin embedding. Histology was performed on sections stained with Hematoxylin & Eosin (H&E). For xenograft experiments, 10^6^ MDA-MB-231 cells were injected subcutaneously in the rear flank of immunocompromised NOD/SCID mice. Tumor growth was monitored, and when tumors reached a palpable and measurable by caliper size (diameter of 1 mm for the early stage and ~3 mm for the advanced stage cohort), mice were assigned randomly in one of three cohorts, vehicle (PBS), soluble DOX (7 mg per kg) and MAG-ARA/DOX, which were administered through tail vein injection in a 100 uL volume^33^. Mice were monitored daily and sacrificed one week later (advanced stage cohort), or injected for the second time on Day 7 and monitored through Day 14, at which point they were sacrificed. Tumors were biopsied and processed as above. Tumor volume was calculated using the formula V = (length/2)*width^2, 34^.

### MR imaging

All MRI experiments were performed in a General Electric Sigma HDxt 1.5 Tesla system^35^. A dual coil consisting of two 10 cm rings set in parallel was used for imaging. All mice were set at close proximity to the two parallel coils and at their center. The body coil of the system was used as transmitter, while the dual coil was used as a receiver. The described set-up was successful towards homogeneous excitation of the sample as well as a high signal-to-noise ratio reception.

The imaged mice were of NOD/SCID strain, with dimensions 10 × 5 × 3 and an average weight of 25 g. All mice were anaesthetized prior to imaging with intraperitoneal injection of 150 μl of Avertin Tribromoethanol as previously described^36–38^. After allowing a time period of 8 minutes for mice to be fully anesthetized they were placed in prone position in a dedicated non-magnetic box. The MRI protocol for imaging mice included fast spin-echo T1, fast spin-echo PD, SPGR, as well as gradient-echo. For the assessment of contrast-to-noise ratio efficiency of each magnetic nanoparticle, T1 MRI measurements were considered, with the following parameters’ values: TE = 14.2 ms, TR = 780 ms, slice thickness = 3 mm, slice gap = 0.3 mm, FOV = 8 × 8 cm, matrix = 256 × 192, plane: axial, number-of-excitations = 3. The total scan time for mice was 15 minutes. Mice were initially scanned without any contrasting agent, and upon completion of the scan mice were injected intravenously (through the tail vein) with 0.2 mL/Kgr Dotarem and rescanned at the exact position. Within three days, mice were injected with MAG-ARA NPs and scanned again. MRI image analysis was performed with the Evorad software. Signal-to-noise ratio (SNR) was calculated with the formula: SNR = 0.655*(ROI_tumor_/averageROI_background_); ROI: region of interest, contrast-to-noise ratio (CNR) for Dotarem and MAG-ARA was calculated by subtracting SNR before and after administration of contrasting agent: CNR = SNR_pre_ − SNR_post_.

## Electronic supplementary material

Supplementary Information
