# Supplementary material for: Magnetic nanoparticles coated with polyarabic acid demonstrate enhanced drug delivery and imaging properties for cancer theranostic applications
Source: Sci Rep. 2017 Apr 10;7:775. doi: 10.1038/s41598-017-00836-y (PMC5429723; doi:10.1038/s41598-017-00836-y)
Supplement: Supplementary file 1 — Supplementary Information [file 41598_2017_836_MOESM1_ESM.pdf]

Supplementary File

Magnetic Nanoparticles Coated with Polyarabic Acid Demonstrate Enhanced Drug Delivery and Imaging Properties for Cancer Theranostic Applications

*Maria Patitsa[1], Konstantina Karathanou[1], Zoi Kanaki[1], Lamprini Tzioga[1], Natassa Pippa[2], Constantinos Demetzos[2], Dimitris A. Verganelakis[3], Zoe Cournia[1] and Apostolos Klinakis[1]**

Protocol for atomistic Molecular Dynamics (MD) simulations

Initially, 50,000 minimization steps using the steepest descent method were performed for the system of the magnetite core attached to the polyarabic acid. Subsequently, MD simulations for 1 ns of the MAG-ARA were performed in the gas phase using constant number of particles, volume and energy (NVE ensemble). The MAG-ARA MNP was then placed in the lipid bilayer using VMD. Subsequently, we carried out Molecular Dynamics (MD) simulations for 50 ns of the whole system in the water phase using constant pressure, temperature, and number of particles (NPT ensemble). The temperature was kept constant at 310 K using the Nose-Hoover thermostat with a relaxation time of 1ps. The Parrinello-Rahman algorithm was used to maintain constant pressure. Electrostatic interactions were calculated with particle mesh Ewald. The real-space electrostatic and van der Waals cutoff distances were set at 12 Å, with smooth truncation starting at 10 Å. A simulation time step of 1 fs and periodic boundary conditions were employed.

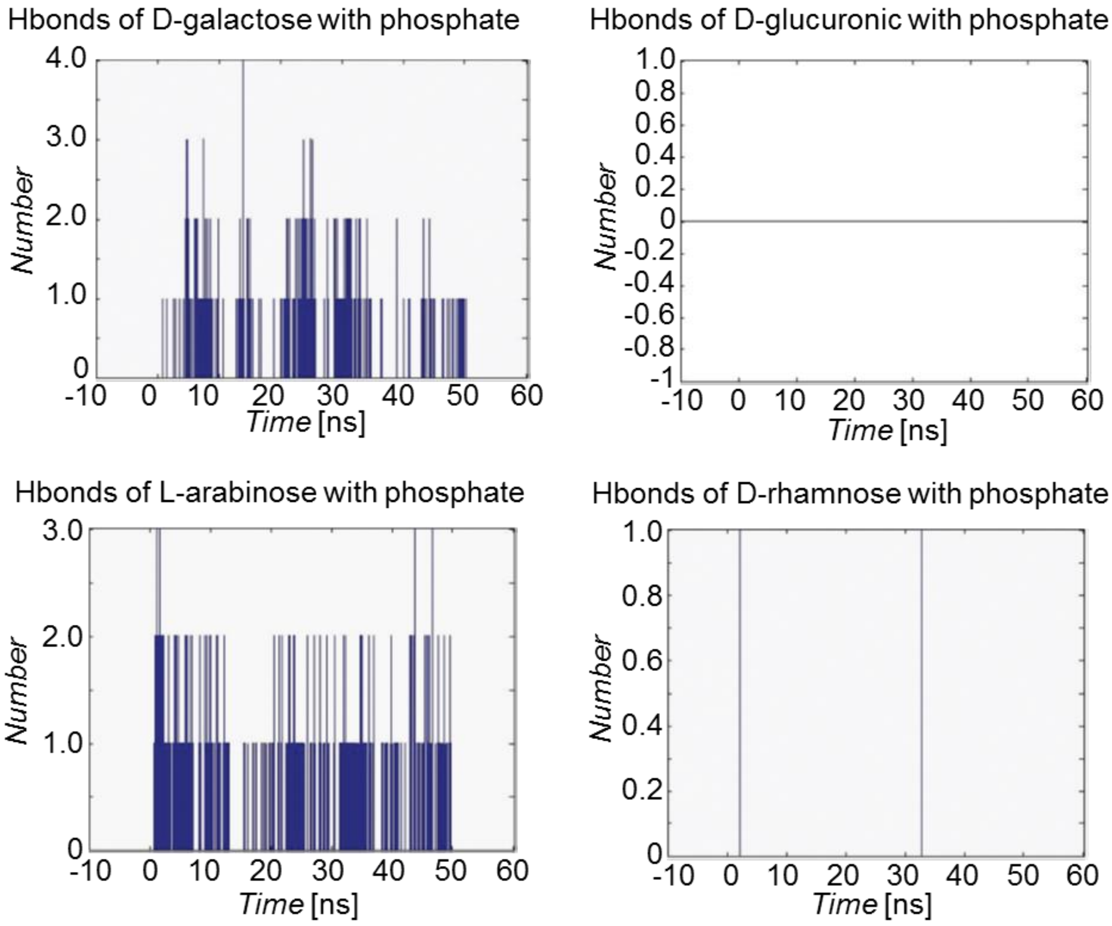

Figure S1. Hydrogen bond analysis between the four different sugars of the polyarabic acid and the phosphate group of the lipid bilayer.

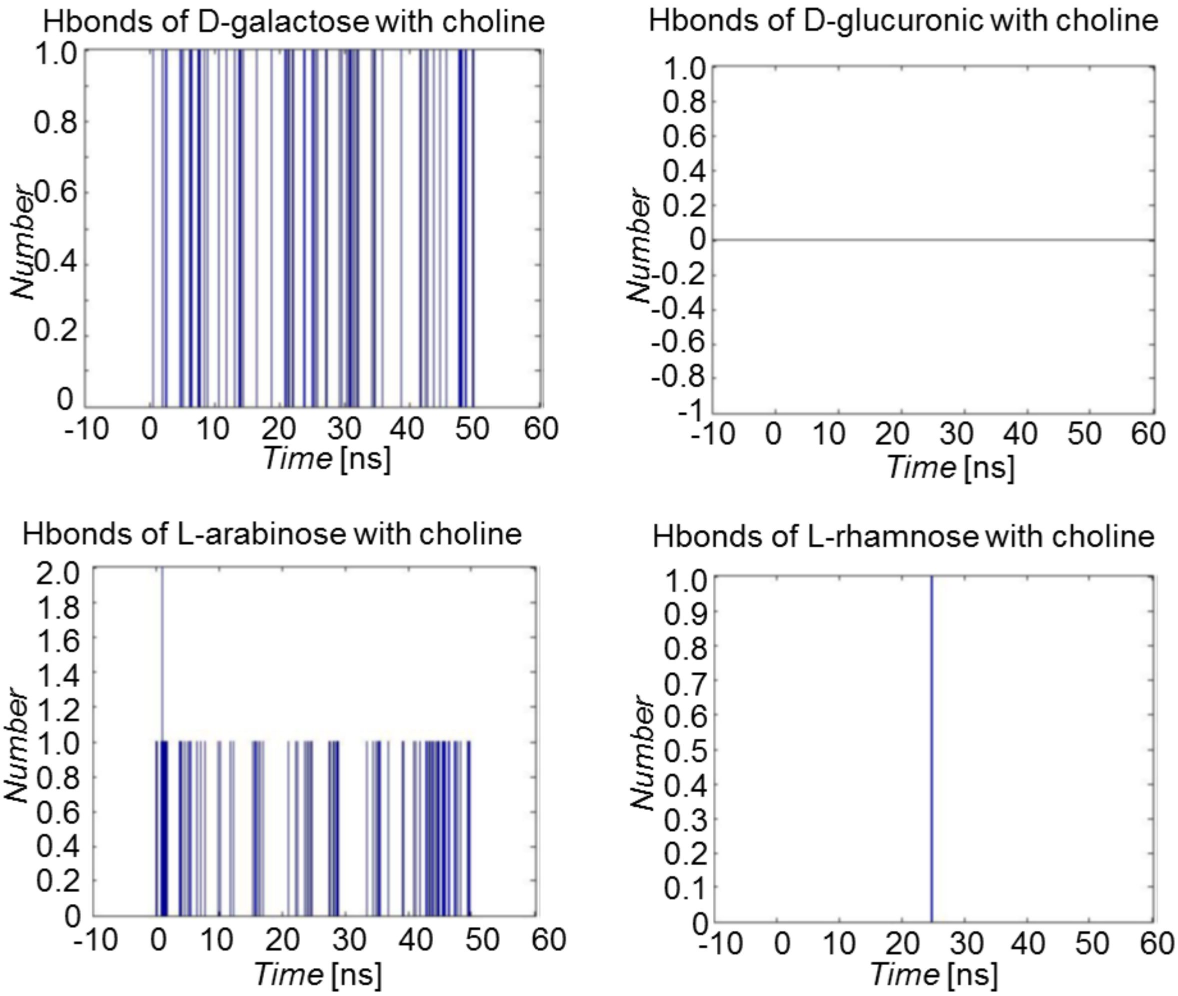

Figure S2. Hydrogen bond analysis between the four different sugars of the polyarabic acid and the choline group of the lipid bilayer.

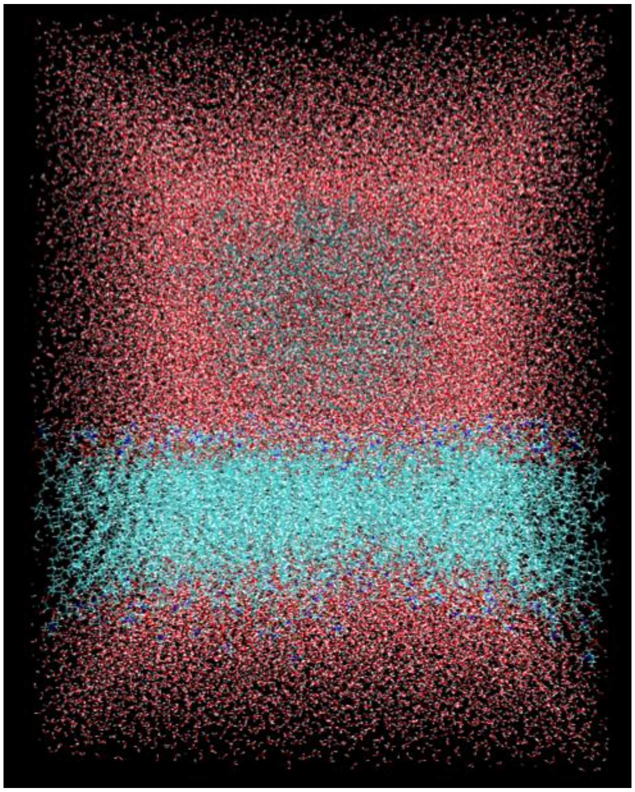

Figure S3. The full simulated system consisting of the functionalized magnetic core with polyarabic acid (MAG-ARA) in contact with a DPPC bilayer and solvated in water.

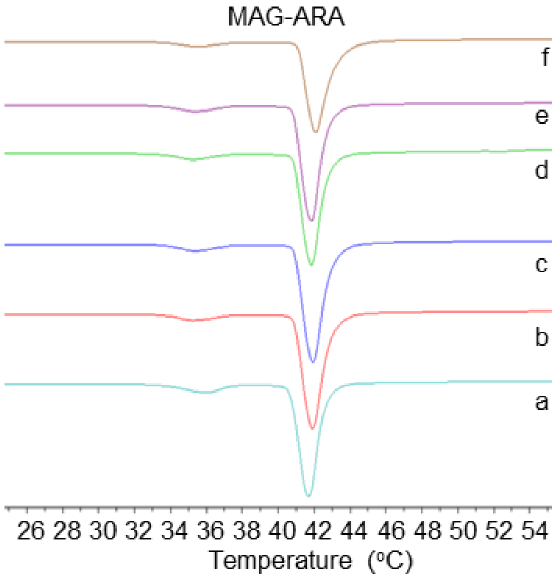

Figure S4. DSC heating of DPPC:MAG-ARA200nm at a. 1:0, b. 200:1, c. 100:1 d. 40:1, e. 10:2 and f. 2:1 w/w. The limits for the calculation of thermotropic parameters are from 25°C to 45°C.
